# Allopregnanolone in Postpartum Depression

**DOI:** 10.3389/fgwh.2022.823616

**Published:** 2022-04-26

**Authors:** Graziano Pinna, Felipe B. Almeida, John M. Davis

**Affiliations:** ^1^The Psychiatric Institute, Department of Psychiatry, College of Medicine, University of Illinois at Chicago, Chicago, IL, United States; ^2^Graduate Program in Health Sciences, Universidade Federal de Ciências da Saúde de Porto Alegre (UFCSPA), Porto Alegre, Brazil

**Keywords:** allopregnanolone, post-partum depression, brexanolone, GABA_A_ receptors, neurosteroid-based therapeutics, rapid-acting antidepressants

## Abstract

Postpartum depression (PPD) is a debilitating psychiatric disorder characterized by a high worldwide prevalence and serious long-term negative outcomes for both mothers and children. The lack of a specific treatment and overreliance on pharmacotherapy with limited efficacy and delayed treatment response has constituted a complication in the management of PPD. Recently, the Food and Drug Administration (FDA) in the USA approved a synthetic formulation of the GABAergic neurosteroid allopregnanolone, administered intravenously (brexanolone) for the rapid, long-lasting and effective treatment of PPD. Hereinafter, we review findings on allopregnanolone biosynthesis and GABA_A_ receptor plasticity in the pathophysiology of PPD. We also discuss evidence supporting the efficacy of brexanolone for the treatment of PPD, which opens a promising new horizon for neurosteroid-based therapeutics for mood disorders.

## Introduction

**Figure 1:**
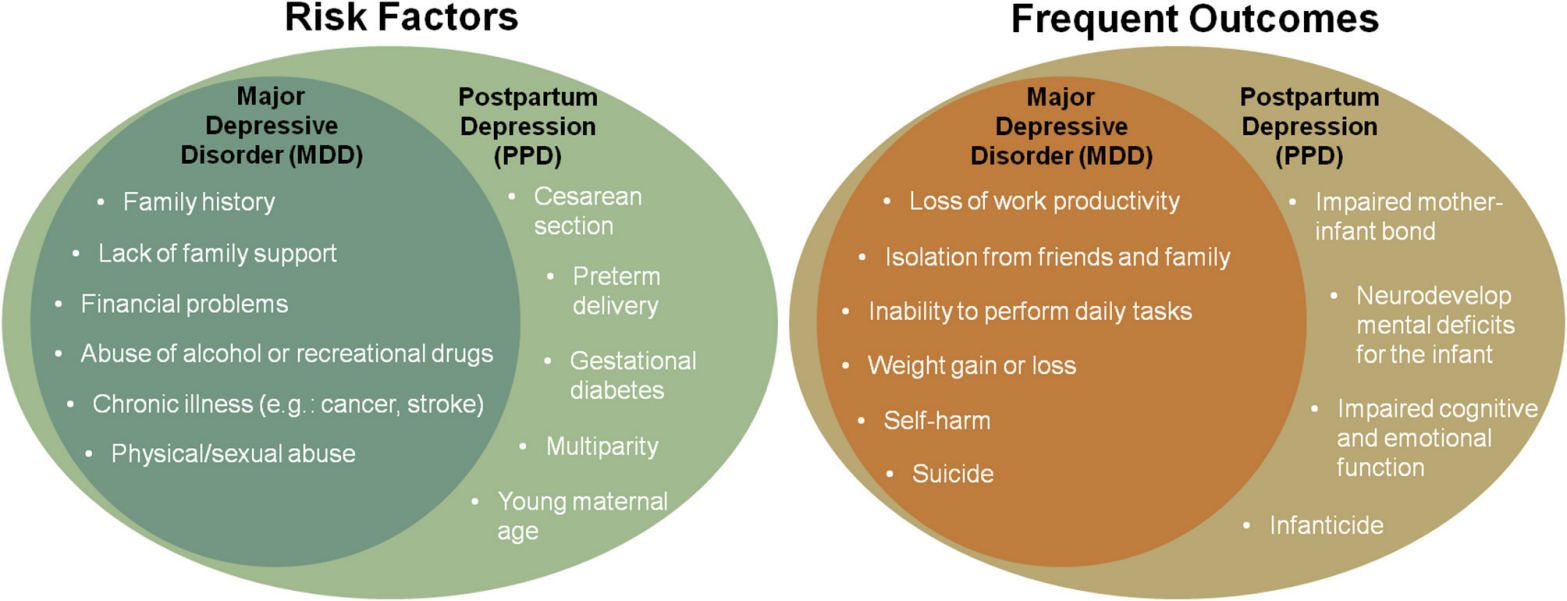
Representation of frequent risk factors and outcomes of major depressive disorder and postpartum depression (PPD).

Postpartum depression (PPD) is a subtype of major depressive disorder that affects around 6.5–12.9% of puerperal women every year (1). The precipitating causes can be similar to major depression and include chronic and acute stress exposure, frequently related to the perinatal period (e.g., gestational diabetes, cesarean section, preterm delivery, teenage pregnancy, lack of social support, sleep disorders, and multiparity). Additionally, past traumatic experiences and stress play a role in the late postpartum onset of PPD (2). PPD is characterized by the emergence, during the postpartum period, of at least one of the core depression symptoms (depressed mood and anhedonia) accompanied by at least five other symptoms, including weight loss, sleep disturbances (insomnia or hypersomnia), psychomotor alterations (agitation or retardation), fatigue, feelings of worthlessness or guilt, impaired concentration, and recurring suicidal thoughts or ideation, for a continuous period of at least 2 weeks and that may last for months and even years. Though the symptomatology of PPD is not differentiable from major depressive disorder, aspects such as symptom severity, heritability, and genetic and epigenetic data suggest that PPD is a distinct condition, particularly when occurring in the early postpartum period (3). In addition to the life-threatening risks imposed on the mother and child (e.g., suicide attempts and infanticide, respectively), the negative impact of PPD on the mother-infant relationship can also be severely disruptive to the newborn in the long term, leading to impaired cognitive and emotional function in adulthood. Frequent risks and outcomes related to major depressive disorder and PPD are represented in Figure 1. For many decades, there was no specific pharmacological therapy approved for PPD treatment. The first-line treatment for this condition includes the antidepressants used in the treatment of the major depressive disorder, such as selective serotonin reuptake inhibitors (SSRIs) (4). Though proven to be effective, SSRIs take several weeks to elicit pharmacological effects and the response rate rarely exceeds 50% (5). Considering that the potential grave consequences of PPD may occur abruptly, fast resolution of symptoms is highly desired. Fortunately, as of 2019, the rapid-acting antidepressant brexanolone (marketed as Zulresso™) has received approval by the United States Food and Drug Administration (FDA) for the treatment of PPD after showing rapid and long-lasting antidepressant effect in the pivotal phase-3 clinical trials (6). Brexanolone is a proprietary pharmaceutical preparation for intravenous (IV) administration of the neurosteroid allopregnanolone. Given allopregnanolone's pleiotropic effects arising from preclinical and clinical studies in the treatment of a large array of neuropsychiatric disorders (7), its approval for the treatment of PPD has generated elevated interest in drug research and development of a new class of therapeutics (8).

This article will focus on the promising new horizon opened by neurosteroid-based treatment for depressive disorders by discussing the role of neurosteroids interfacing with GABA_A_ receptor function in the pathophysiology and treatment of PPD.

## Neurosteroids and GABA_A_ Receptor Plasticity in PPD

Stress plays a major role in both the presentation and severity of PPD and the inability to shut down the stress-induced hypothalamus–pituitary–adrenal (HPA) axis activation has been traditionally suggested as an underlying neurobiological mark of PPD. The GABAergic signaling modulated by neurosteroids, including allopregnanolone and its equipotent GABAergic isomer, pregnanolone, may play a role in some of this disorder manifestation (6, 9, 10). Allopregnanolone also plays a pivotal physiological role by protecting the maternal and fetal brain from harmful levels of maternal glucocorticoids resulting from stress exposure during pregnancy and prevents premature secretion of oxytocin associated with preterm birth. Allopregnanolone is also neuroprotective and promotes the development of the fetal brain (11).

The biosynthesis of allopregnanolone and pregnanolone has been associated with the emergence of depressive disorders and post-traumatic stress disorder (PTSD) (12–14). Allopregnanolone and pregnanolone show remarkable anxiolytic and antidepressant effects, both in humans and in preclinical models (15–17). In 1998, Uzunova et al. (13) and Romeo et al. (12) and respective collaborators simultaneously published that the cerebrospinal fluid (CSF) and serum levels of allopregnanolone were decreased in depressed patients and could be upregulated by SSRI antidepressant treatment. Specifically, allopregnanolone concentration in the CSF correlated with the severity of depressive symptoms, and treatment with fluoxetine and fluvoxamine increased its content only in patients who responded to the treatment with remission of depressive symptoms (12, 13). These studies were followed by findings that allopregnanolone levels decreased similarly in women and men with PTSD, with the lowest levels found in subjects exhibiting PTSD with comorbid depression (14, 18, 19). Ratios of allopregnanolone with precursors and enzymatic expression analysis have suggested a sex-dependent dimorphism in the enzymes that regulate neurosteroid biosynthesis (20). In the post-mortem brain of depressed subjects, allopregnanolone appeared to be decreased by a 5α-reductase type I (5α-RI) expression deficit in the prefrontal cortex Brodmann's area 9 (BA9) (21). Ratio analysis of progesterone to 5α-dihydroprogesterone, and from this to allopregnanolone in PTSD subjects, indicated that while in men, allopregnanolone decreased from a 5α-RI deficit, in women, the enzyme 3α-hydroxysteroid dehydrogenase appeared to be affected (22). Preclinical studies in rodent models of PTSD and depression have confirmed these clinical findings showing altered neurobiology in tests reproducing affective symptoms in humans (23, 24). These findings have implicated allopregnanolone in the pathophysiology of PTSD and depressive disorders, which led to the proposal of its potential biomarker role for subtypes of mood disorders [reviewed in (20, 24, 25)].

There has been limited investigation on neurobiological mechanisms underlying behavioral predictors of PPD during pregnancy and the postpartum period. Although a disorder characterized by a distinct phenotype from major depressive disorder, PPD is also characterized by changes in GABA_A_ receptor neurotransmission, including altered expression of the receptor subunits and impaired neurosteroid biosynthesis (26–28). However, transient postpartum hypothalamic corticotropin-releasing hormone (CRH) suppression (26), in conjunction with the steroid withdrawal in the aftermath of parturition, is often regarded with the affective instability observed during the postpartum period (6). Even though there is a coincidental timing of abrupt neuroactive steroid decline—including progesterone and allopregnanolone—after parturition and the onset of PPD symptoms, investigations that have attempted to demonstrate lower allopregnanolone levels in the postpartum period in subjects with PPD have often produced mixes results.

Luisi et al. (29) investigated allopregnanolone and progesterone concentrations in maternal and cord serum by radioimmunoassay (RIA). Their concentrations steadily increased throughout the gestation period. At delivery, their serum levels were significantly lower in women who underwent emergency cesarean section. Similarly, umbilical cord serum allopregnanolone and progesterone levels were decreased in an emergency cesarean than in vaginal delivery. Intriguingly, in subjects with chronic hypertension, serum allopregnanolone concentration was significantly increased when compared with the levels in healthy women. In another study conducted in healthy volunteers with low and high psychological scores assessed by the SCL-90 psychometric scale, neuroactive steroids were also measured by RIA during the follicular phase (FP), the luteal phase (LP), and at four time points during pregnancy. Progesterone and allopregnanolone levels were higher in LP than in FP and they consistently increased with the progression of pregnancy, however, without differences between low and high psychological score groups (30). Peripartum plasma levels of neuroactive steroids and GABA were quantified by liquid chromatography-mass spectrometry (LC-MS) in healthy subjects and subjects at-risk for PPD established by a prior history of depression or who showed mild depressive or anxiety symptoms. Peripartum GABA levels were lower and progesterone and pregnanolone levels were higher in at-risk PPD vs. healthy subjects. Trait-anxiety scores were positively associated with pregnanolone and allopregnanolone (31). In another study, the authors hypothesized that peripartum neuroactive steroids are related to resting-state functional connectivity in PPD compared to healthy subjects. Plasma allopregnanolone was elevated in subjects with PPD and positively correlated with dorsomedial prefrontal cortex (DMPFC) connectivity in women at risk for PPD (32). In a prospective, nested, case-control study in low-income women of color in early pregnancy, Wenzel et al. (33) examined the concentrations of progesterone, as well as allopregnanolone and pregnanolone and the levels of the allopregnanolone isomers, isoallopregnanolone and epipregnanolone, which act as negative allosteric modulators of the GABA_A_ receptor. Pregnant women manifested with depression at either or both first and second trimesters. Prenatal depression cases showed higher ratios of both allopregnanolone and pregnanolone to progesterone compared to controls. Subjects with depression at both first and second trimesters showed an increase in epipregnanolone to progesterone ratios from the first to the second trimester, while control subjects showed a decrease in these ratios. Isoallopregnanolone was found to increase in the second trimester alone. Although associated with an increase of allopregnanolone levels, the increase of allopregnanolone isomers with antagonistic function at GABA_A_ receptors is intriguing and deserves further investigation.

In contrast to these investigations, other studies successfully observed lower allopregnanolone levels in association with anxiety and depression symptoms. Mood and anxiety and allopregnanolone were examined across the peripartum by ELISA at the second and third trimesters and week 6 postpartum in women with a history of mood and/or anxiety disorders and healthy controls. Lower allopregnanolone levels at the postpartum period were associated with higher depression and anxiety scores. This exploratory finding suggests that the relationship between allopregnanolone and mood and anxiety symptoms may change across the peripartum (9). Serum allopregnanolone levels were found significantly lower in women manifesting postpartum “blues” when compared to euthymic women, while progesterone levels did not differ significantly. A significant negative correlation was observed between the Hamilton score and levels of serum allopregnanolone and progesterone (34).

A study that quantified serum allopregnanolone by Celite chromatography and RIA found that women who had elevated depression scores also had significantly lower allopregnanolone levels compared to healthy subjects. Furthermore, a significant negative correlation was observed between self-rated depression scores and allopregnanolone serum concentrations. Self-rated anxiety was not associated with allopregnanolone serum concentrations during pregnancy. This study supports that high allopregnanolone concentrations may underlie depressed mood during pregnancy (35). In another study that examined second and third trimester progesterone and allopregnanolone levels by ELISA, while PPD was diagnosed by clinician interview in pregnant women who had prior diagnosis of mood disorders, it was observed that every additional ng/ml of second trimester allopregnanolone resulted in a 63% reduction in the risk of developing PPD (9).

Association among stress-related neurobiological factors (GABAergic neurosteroids) and indices of anxiety during pregnancy showed that lower progesterone and combined measures of allopregnanolone + pregnanolone were associated with greater negative emotional responses to stress, and lower cortisol was associated with worse sleep quality. These data suggest that progesterone and allopregnanolone + pregnanolone levels in the second trimester of pregnancy are inversely related to negative emotional symptoms, and acute stress challenges appear to reduce these steroids to promote negative emotional responses (36).

Finally, a study demonstrated altered sensitivity to neuroactive steroids specifically in patients presenting a history of PPD (37). This investigation supports that while neuroactive steroid levels may not be abnormal, sensitivity to neuroactive steroids may provide a better explanation for the increased susceptibility to develop risk to PPD. Virtually no studies have investigated this topic in clinical studies, however, several basic research investigations backed this assumption [reviewed in (26)].

These studies (summarized in Table 1) suggest that while exogenous administration of allopregnanolone is an effective treatment to relieve PPD symptoms, the comprehension of the mechanisms linking neuroactive steroid levels with the onset of symptoms remain elusive. Most of these studies have used an array of different technologies to quantify neuroactive steroids. Hence, procedural methodologies in computing neuroactive steroid analyses may also play a role in explaining the discrepancy in these results. It is also important to note that deficits in allopregnanolone biosynthesis play an important role in major depressive disorder pathophysiology, in addition to their role in the manifestation of depression symptoms during pregnancy [reviewed in (24)].

**Table 1:** Summary of studies investigating allopregnanolone levels during pregnancy and its relationship with postpartum depression (PPD).

**References**	**Allopregnanolone levels during pregnancy (except where otherwise stated)**	**Matrix**	**Method of measurement**
Luisi et al. (29)	Increased progressively; at delivery, levels were significantly lower in women who underwent emergency cesarean section		RIA
Paoletti et al. (30)	Increased progressively in both women with high and low psychological score assessed by the SCL-90 psychometric scale	Serum	RIA
Deligiannidis et al. (31)	Higher in women at risk for PPD	Plasma	LC-MS
Deligiannidis et al. (32)	Higher in women with PPD	Plasma	LC-MS
Wenzel et al. (33)	Higher ratio of allopregnanolone to progesterone in prenatal depressed women	Serum	GC-MS
Osborne et al. (9)	Lower allopregnanolone in the 2nd trimester correlated with higher risk of developing PPD	Plasma	ELISA
Nappi et al. (34)	Levels were lower after delivery in women manifesting postpartum “blues”	Serum	RIA
Hellgren et al. (35)	Lower in women with elevated depression scores	Serum	RIA
Crowley et al. (36)	Lower levels were associated with greater negative emotional responses to stress	Serum	GC-MS

*RIA, radioimmunoassay; LC-MS, liquid chromatography-mass spectrometry; GC-MS, gas chromatography-mass spectrometry; ELISA, enzyme-linked immunoassay*.

Changes in GABA_A_ receptor subunit expression have been demonstrated during protracted stress conditions and in the pathophysiology of PPD in both preclinical and clinical studies. In peripheral blood mononuclear cells, the expression of δ and ρ2 subunits was upregulated during pregnancy in a clinical study (27). Maguire and Mody (28) observed a decrease of both δ and γ2 subunits in a mouse model of PPD, which resulted in decreased tonic and phasic inhibition in pregnant mice. Specifically, the δ subunit expression changes were associated with depressive-like phenotypes and abnormal maternal behaviors. In a rat pregnancy model, the cerebral cortex and hippocampus expression of the GABA_A_ receptor γ2 subunit decreased during pregnancy, before returning to baseline levels 2 days after delivery. These data were further validated in a model of 5α-reductase (the rate-limiting step-enzyme in allopregnanolone biosynthesis) blockade in pregnant rats, which reduced both plasma and brain allopregnanolone content and prevented the decrease of γ2 mRNA expression observed during pregnancy. Furthermore, these subunit changes resulted in structural and functional changes in the GABA_A_ receptor demonstrated by decreased stimulatory effect of the GABAmimetic drug muscimol on Cl^−^ uptake by cerebrocortical membranes. These observations support a role for allopregnanolone in regulating the plasticity of GABA_A_ receptor-containing γ2 subunit during pregnancy and after delivery (38). Of note, changes in GABA_A_ receptor subunit expression are also affected by protracted stress in rodent models of anxiety and depression. Decreased γ2 and increased α4 GABA_A_ receptor subunit expression were observed in the hippocampus and frontal cortex, which correlated with altered pharmacological response to sedative and anxiolytic effects of benzodiazepines (39, 40). Another study in stressed rats also showed upregulation of hippocampal α4 and δ subunits (41). A switch among extrasynaptic δ subunit and synaptic γ2 subunits was observed during pregnancy and across the estrous cycle (38, 42).

Collectively, these studies suggest that impaired dynamic reconfiguration of GABA_A_ receptor subunit subtypes, their sensitivity to neuroactive steroids, and neuroactive steroid biosynthesis during the perinatal period deserve further investigation.

## Brexanolone Effects in the Treatment of Postpartum Depression

The finding that allopregnanolone is decreased in subjects with depression and that SSRIs restore allopregnanolone to normal concentrations in treatment responders has stimulated studies to understand the underlying mechanisms of the neurosteroidogenic effects of these compounds in relieving symptoms of depression and led to exploit neurosteroidogenic targets as novel paths to treat mood disorders (43). In rodent stress models of neurosteroid biosynthesis downregulation and behavioral traits of mood disorders (anxiety-like, aggressive, depression-like behavior, deficits in fear extinction), Pinna et al. investigated the steroidogenic action of SSRIs (44). They found that SSRIs upregulate allopregnanolone by a mechanism independent of serotonin reuptake inhibition, suggesting SSRIs act specifically as selective brain steroidogenic stimulants (SBSSs) at effective doses that are one level of magnitude lower than the active SSRI doses. Intriguingly, behavioral improvement occurred very rapidly (hours) and was long-lasting (45). Clinical trials testing the hypothesis that IV allopregnanolone supplementation could offer a strategy to improve PPD showed improvement in symptoms within hours of active versus placebo infusion (46–48). Four clinical trials assessed the antidepressant efficacy of brexanolone infused over 60 h in women recruited between 6 weeks and 6 months postpartum. In a proof-of-concept study, open-label clinical trial, four women who developed severe PPD received 60 mcg brexanolone infusions. Safety, efficacy, and tolerability assessed by this study showed that all patients were able to complete the infusions, and after 60 h, the depression symptoms dramatically decreased. Sedation accounted for the most severe adverse effects accompanied by pain, rash, dizziness, and flushing. Elevated cost also contributes to an important limitation of brexanolone IV treatment, and an oral formulation of allopregnanolone could abate drug cost and avoid hospitalization required during the IV administration. In phase 3, a double-blind, randomized, outpatient, placebo-controlled clinical trial, an oral formulation of allopregnanolone (e.g., zuranolone) was investigated in women with PPD (49). Zuranolone administered at the dose of 30 mg for 2 weeks significantly improved the Hamilton depression rating scale (HAMD-17) scores 3 days after administration, and this effect was maintained for a 2-week treatment and 45-day follow-up vs. placebo. While a clinical study to investigate the efficacy of zuranolone for major depression showed mixed results, this randomized clinical trial showed improvement of depression with minimal side effects, thereby supporting the development of zuranolone in the treatment of PPD and major depression. Importantly, even though a direct comparison between the effectiveness of the treatment with allopregnanolone vs. that of SSRIs has not been evaluated, the clinical efficacy of brexanolone appears superior to that of widely prescribed traditional antidepressants (22).

While several mechanisms remain to be further investigated, collectively, these studies provide strong support for allopregnanolone biosynthesis and GABA_A_ receptor sensitivity disturbances in underlying PPD pathophysiology and support the development of neurosteroid-based treatment for rapid improvement of mood disorders (50).

## Author Contributions

GP conceptualized and wrote the manuscript. GP, FA, and JD revised the manuscript draft. All authors contributed to the article and approved the submitted version.

## Funding

FA received a Doctoral Dissertation Research Award from the Fulbright Commission Brazil.

## Conflict of Interest

GP is a paid consultant to PureTech Health (Boston, MA, USA), GABA Therapeutics, and NeuroTrauma Sciences (Alpharetta, GA, USA). He has two patent applications, one on N-palmitoylethanolamine (PEA) and peroxisome proliferator-activated receptor alpha (PPAR-α) agonists US20180369171A1, pending, and one on allopregnanolone analogs US11266663B2 granted on March 8, 2022 in the treatment of neuropsychiatric disorders. The remaining authors declare that the research was conducted in the absence of any commercial or financial relationships that could be construed as a potential conflict of interest.

## Publisher's Note

All claims expressed in this article are solely those of the authors and do not necessarily represent those of their affiliated organizations, or those of the publisher, the editors and the reviewers. Any product that may be evaluated in this article, or claim that may be made by its manufacturer, is not guaranteed or endorsed by the publisher.
